# Alcohol Use, Risky Alcohol Use, and Associated Factors Among Adults Living with HIV in Urban Dar es Salaam, Tanzania

**DOI:** 10.1007/s10461-026-05094-6

**Published:** 2026-03-10

**Authors:** Belinda J. Njiro, Jackline E. Ngowi, Joel M. Francis, Till Bärnighausen, Pascal Geldsetzer, Wafaie W. Fawzi, Muhammad Bakari, Christopher R. Sudfeld, Bruno F. Sunguya

**Affiliations:** 1 Department of Epidemiology and Biostatistics, School of Public Health and Social Sciences, Muhimbili University of Health and Allied Sciences, Dar es Salaam, Tanzania; 2 Population Health Sciences Institute, Bristol Medical School, University of Bristol, Bristol, England; 3 Department of Community Health, School of Public Health and Social Sciences, Muhimbili University of Health and Allied Sciences, Dar es Salaam, Tanzania; 4 Department of Family Medicine and Primary Care, School of Clinical Medicine, Faculty of Health Sciences, University of the Witwatersrand, Johannesburg, South Africa; 5 Heidelberg Institute of Global Health, Heidelberg, Germany; 6 Division of Primary Care and Population Health, Department of Medicine, Stanford University, Stanford, CA USA; 7 Department of Global Health and Population, Harvard T. H. Chan School of Public Health, Boston, MA USA; 8 School of Clinical Medicine, Muhimbili University of Health and Allied Sciences, Dar es Salaam, Tanzania

**Keywords:** Alcohol use, Risky alcohol drinking, People living with HIV, AUDIT-C, Tanzania

## Abstract

Alcohol use and risky alcohol use are associated with health, social, and psychological complications and may interfere with HIV/AIDS treatment. This study assessed the prevalence and factors associated with alcohol use and risky alcohol use among adults living with HIV in Dar-es-Salaam, Tanzania. This cross-sectional study included data from 771 adults living with HIV on antiretroviral therapy (ART) who were enrolled in a non-inferiority cluster randomized controlled trial. Alcohol use and risky drinking in the past 12 months were assessed using the Alcohol Use Disorders Identification Test (AUDIT-C) tool. Log-binomial regression models were applied to identify factors associated with alcohol use and risky alcohol use. Overall, 31.4% of participants reported current alcohol use (*n* = 242). Among these individuals, 45.5% (*n* = 110) engaged in risky alcohol use (14.2% among all participants). About a quarter (23.2%) were classified as engaging in heavy episodic drinking (HED). In multivariable models, older adults (RR = 0.27; 95%CI: 0.13–0.54) and males (RR = 0.68; 95%CI: 0.46–1.01) had a lower risk of risky alcohol use. Individuals who had disclosed their status to their partners were more likely to report risky alcohol use compared to those who had not disclosed (RR = 1.33; 95%CI:1.00–1.78). These findings indicate that alcohol use is common among adults living with HIV, with half of current drinkers engaging in risky consumption patterns, including HED. Risky alcohol use was more prevalent among younger adults and women. These results underscore the need for targeted interventions addressing risky alcohol use within primary HIV care settings, particularly for young adults and women.

**Supplementary Information:**

The online version contains supplementary material available at https://doi.org/10.1007/s10461-026-05094-6.

## Introduction

Alcohol use disorders (AUD) are among the most prevalent mental health disorders worldwide [1]. In 2016, 5.1% of people globally were reported to use alcohol [2]. The prevalence of alcohol use globally is also estimated to be five times higher among men compared to their female counterparts. Among people living with HIV (PLHIV), the pooled prevalence of AUD worldwide is 29.8%, and it is twice as high in males compared to females [3]. Nevertheless, alcohol use has been increasing over time for both sexes globally [4]. HED, also known as binge drinking, is another concept as part of risky alcohol use defined as consumption of 60 g or more of pure alcohol, which has been equated to consumption of six or more standard alcohol drinks in the past 30 days [5, 6]. Risky alcohol drinking, including HED are major risk factor for various health outcomes, including non-communicable diseases (NCDs) such as cardiovascular diseases, liver cirrhosis and cancers. Prenatal alcohol exposure can lead to neurodevelopmental problems in newborns [7]. Psychosocially, alcohol problems and binge drinking have been linked to poor mental health [8] and reported to co-exist with psychiatric disorders such as depression, anxiety, personality disorders, and general substance use disorders [9].

Compared to the general population, higher levels of alcohol use have been consistently reported among people living with chronic conditions, increasing their risk of morbidity and mortality [10]. Alcohol use and hazardous drinking are associated with health, social, and psychological complications in the general population and may particularly interfere with the treatment of chronic conditions, including HIV/AIDS [11]. Among other factors, alcohol use may interfere with antiretroviral therapy (ART) adherence in the context of HIV, which may lead to virological non-suppression and negative health consequences [12]. In sub-Saharan Africa (SSA), PLHIV who reported to be at-risk drinkers had a two to four times higher odds of ART nonadherence compared to social drinkers [12–14]. Similarly, harmful alcohol use is associated with a higher likelihood of viral non-suppression and treatment failure, with evidence showing the mediated effect of ART adherence in this relationship [12, 15].

Risky alcohol use is associated with high-risk sexual behaviors that may further increase the risk of HIV and STI transmission [16]. PLHIV with a reported history of alcohol use are also at a higher risk of progressing to risky drinking and AUD compared to individuals without HIV [17, 18]. Further impacts of AUD in PLHIV can include isolation and decreased care-seeking behaviors [19], greater risk of HIV/AIDS disease progression, and increased risk of opportunistic infections [20]. People with hazardous alcohol use may also have lower ART adherence due to fear of the interaction between ART drugs and alcohol, which may also increase the risk of HIV drug resistance [21, 22].

Currently, most lower and middle-income countries, including Tanzania, are working towards achieving the UNAIDS 95-95-95 goals, especially the third 95% target focused on virologic suppression [13]. In Tanzania, 94% of PLHIV on ART and 78% among all PLHIV have suppressed viral load. While Tanzania has achieved the second target at 98%, the country is still lagging in HIV diagnosis and viral load suppression. Multiple factors have been reported to affect virological suppression and treatment outcomes for PLHIV, including gender, socioeconomic status, treatment adherence, opportunistic infections, WHO stage, and substance use [23–25]. Identification of context-specific factors hindering the attainment of the remaining targets in Tanzania is warranted.

There is limited evidence on the burden and individual-level factors associated with alcohol use and risky alcohol use among PLHIV in urban SSA settings. Recent evidence on the burden of AUD in PLHIV has focused on rural settings which may have different exposures and contexts compared to urban settings [26]. This study aimed to determine the prevalence of alcohol use and risky alcohol use and associated factors among PLHIV in an urban setting of Dar es Salaam, Tanzania.

## Methods

### Study Design and Setting

In this cross-sectional study, we analyzed the baseline data from a completed non-inferiority cluster randomized controlled trial that was implemented in the three municipalities of the Dar es Salaam region in the United Republic of Tanzania. The parent trial aimed to assess the feasibility and effectiveness of community health workers (CHW)-led home delivery of ART in the public-sector healthcare system in Dar es Salaam, Tanzania [27]. The intervention was based on an ART differentiated care model that employed home-based ART delivery for clinically stable patients on ART and facility-based care for those who were not stable on ART. Forty-eight clusters (healthcare facilities) in three districts were randomized to either the differentiated model or standard of care (facility-based care for all ART patients). Participants were enrolled from February 2016 to January 2017 and followed up for 12 months [27]. Data collection was conducted at the respective facilities by trained data collectors through provider-assisted tablet-based questionnaires. The current analysis only includes data collected from the baseline phase of the trial.

### Study Population

Participants were eligible for inclusion in the parent trial if they were aged 18 years or older, were attending one of the participating healthcare facilities for ART, and were living in a neighborhood in the facility’s catchment area [27].

After assessing full eligibility, 2172 adults aged 18 years or older living with HIV who attended any of the 48 HIV care and treatment facilities (CTC) in Dar es Salaam, Tanzania were eligible to proceed with randomization. Due to loss to follow-up, death, patient transfer, and data linkage challenges, only 1,815 were analysed in the primary trial. Participants in the intervention facilities were required to be clinically stable on ART. Clinical stability was defined as virological suppression from the most recent viral load taken within 12 months, or a CD4-cell count > 350 cells/µL taken in the 12 months prior to enrolment. This criterion did not apply to participants in the control arm facilities. The study excluded participants who were pregnant or were unable to provide consent. Data collection was conducted in two phases; alcohol use-related questions were only collected in the latter phase following protocol amendments. The current analyses only covered 771 participants who responded to AUDIT-C questions in both intervention and control arms. No significant differences were observed between responders of the AUDIT-C questions and non-responders (Supplementary Table 1).

### Data Collection and Study Variables

Alcohol use and risky drinking were assessed using the Alcohol Use Disorders Identification Test (AUDIT-C) administered at baseline of the parent trial. The AUDIT-C tool is a brief screening test for problematic or risky alcohol drinking [28]. It has been adopted and previously used in the Tanzanian context with acceptable psychometric properties [11, 29]. The tool determines risky alcohol use using three sets of questions for participants reporting alcohol use in the last 12 months. All the questions were rated on a five-point Likert scale as follows:

1. How often do you have a drink containing alcohol? Responses: Monthly or less, two to four times a month, two to three times per week, and four or more times a week.
2. How many standard drinks do you have in a typical day when drinking? Responses: 1 or 2 drinks, 3 or 4 drinks, 5 or 6 drinks, 7–9 drinks, and 10 or more.
3. How often do you have six or more standard drinks on one occasion? Responses: Never, less than monthly, monthly, weekly, daily or almost daily.

The total AUDIT-C scores were generated based on participants’ responses, and a score of 3 or more for females and 4 or more for males was considered risky alcohol use [28]. The third question of the AUDIT-C tool was used in assessing heavy episodic drinking (HED) in this study. HED was assessed as the consumption of the equivalent of 6 + drinks per occasion at least monthly [5].

### Covariates

A semi-structured provider-assisted questionnaire was used to collect data on participant socio-demographic characteristics, self-reported ART adherence, duration since HIV diagnosis, duration on ART, and HIV disclosure status. ART adherence was assessed using a single question asking participants to rate their adherence to ART in the last month. The score was reported in a Likert scale from 1 as very poor to 6 as excellent. For participants without recent CD4 + counts data, whole blood samples were collected for assessment at baseline and endpoint. Pre-recorded CD4 + measured within 12 months before the study enrollment were used as baseline CD4 + counts [27].

### Data Management and Analysis

We first described characteristics of the study population with means or medians and proportions. The prevalence of alcohol use and risky alcohol use was determined among participants of the trial who completed the AUDIT-C questions on alcohol use.

To determine factors associated with risky alcohol use, we conducted univariate and multivariable negative log-binomial regression and estimated relative risks (crude and unadjusted analyses). We computed two models, the primary model assessed factors associated with risky alcohol use among participants who reported alcohol use in the past 12 months (*N* = 242). The second model assessed factors associated with risky alcohol use among all PLHIV in our analysis (*N* = 771). Multivariable models included independent variables associated with risky alcohol use, with p-value < 0.2 in the univariable models. Age, sex, HIV status disclosure to partners/spouse, time since HIV diagnosis, and ART adherence status were included as apriori-decided variables based on previous evidence of their role on alcohol use [17, 26, 30–32]. Age was categorized into three groups: Young adults (18–24 years), adults (25–49 years), and older adults (> 49 years). CD4 counts were categorized as stage 1 (at least 500 cells/ul), stage 2 (200–499 cells/ul), and stage 3 or advanced HIV disease (< 200 cells/ul) [33]. Risk ratios (RR) and corresponding 95% confidence intervals were calculated, and a p-value of < 0.05 was considered statistically significant. The missing indicator method was used to retain data from individuals missing covariate data [34]. All analyses were computed using STATA software version 17 [35].

### Ethical Considerations

The study received ethical approval from the National Health Research and Ethics Committee (NatHREC) at the National Institute of Medical Research (NIMR), Tanzania, reference number NIMR/HQ/R.8a/Vol. IX/1989. It received an exemption from the Harvard T.H. Chan School of Public Health institutional review board in June 2015. All participants provided written informed consent during enrolment and before administering the study exit questionnaires.

## Results

Of the 2172 eligible adults recruited in the primary trial, only 1,815 were analysed due to loss to follow-up, death, patient transfer, and data linkage challenges. The current analysis included data from 771 adults living with HIV who responded to alcohol use questions; characteristics of the study population are presented in Table 1. Most participants were females (82.5%, *n* = 636), with a slightly higher proportion of males among participants reporting alcohol use compared to non-users (21.5% vs. 15.7%) and middle-aged adults within the 25–49 age band (83.1%, *n* = 641), with a comparable age distribution across alcohol use groups. The mean age (standard deviation (SD)) was 38 (8.4) years. Half of the participants were currently married or were living with partners (48.1%, *n* = 371); compared to non-users, participants who reported alcohol use were slightly less likely to be married. Employment was more common among those who use alcohol than non-users (91.3% vs. 82.6%). Almost half of the participants were on ART for more than a year (47.5%, *n* = 366). Approximately 4.2% (*n* = 32) of participants had a CD4 count of 200 cells/µL or less. A greater proportion of participants who reported alcohol use had CD4 counts ≥ 500 cells/µL compared to non-users (39.7% vs. 25.7%). HIV status disclosure to anyone was high in both groups (over 90%), yet more than half had not disclosed to a spouse or partner. ART adherence was generally good to very good (92.2%), though poor or moderate adherence was more prevalent among non-users than those with current alcohol use (7.2% vs. 3.3%).

**Table 1:** Characteristics of the study population of adults living with HIV in Dar es Salaam with data available on alcohol drinking (*N* = 771)

Variables	Alcohol use		Total
	Yes (*N* = 242) *n* (%)	No (*N* = 529) *n* (%)	*N* = 771
Sex			
Female	190 (78.5)	446 (84.3)	636 (82.5)
Male	52 (21.5)	83 (15.7)	135 (17.5)
Age groups – Mean (SD)	38 (8.4)	40 (9.6)	39 (9.2)
18–24	8 (3.3)	23 (4.3)	31 (4.0)
25–49	210 (86.8)	431 (81.5)	641 (83.1)
>49	24 (9.9)	75 (14.2)	99 (12.8)
Education level			
No formal education	12 (5.0)	21 (4.0)	33 (4.3)
Pre-school to college	228 (94.2)	504 (95.3)	732 (94.9)
Unknown	2 (0.8)	4 (0.8)	6 (0.8)
Marital status			
Single/never married	72 (29.8)	146 (27.6)	218 (28.3)
Currently married/living with partner	106 (43.8)	265 (50.1)	371 (48.1)
Previous married/lived with partner	61 (25.2)	113 (21.4)	174 (22.6)
Unknown	3 (1.2)	5 (0.9)	8 (1.0)
Employment status			
Yes	221 (91.3)	437 (82.6)	658 (85.3)
No	21 (8.7)	92 (17.4)	113 (14.7)
Duration since HIV diagnosis - Median (IQR)	3 (2, 6)	3 (2, 7)	3 (1, 7)
<2 years	38 (15.7)	79 (14.9)	117 (15.2)
2–5 years	46 (19.0)	82 (15.5)	128 (16.6)
>5 years	33 (13.6)	85 (16.1)	118 (15.3)
Unknown	125 (51.7)	283 (53.5)	408 (52.9)
Duration on ART- Median (IQR)	29 (12, 47)	32 (15, 63)	32 (14, 63)
<6 months	23 (9.5)	43 (8.1)	66 (8.6)
6–12 months	17 (7.0)	22 (4.2)	39 (5.1)
>12 months	117 (48.3)	249 (47.1)	366 (47.5)
Unknown	85 (35.1)	215 (40.6)	300 (38.9)
CD4 counts (cells/µL) - Median (IQR)	9999 (466, 99999)	671 (376, 99999)	815 (388, 99999)
<200	9 (3.7)	23 (4.3)	32 (4.2)
200-<500	30 (12.4)	61 (11.5)	91 (11.8)
>=500	96 (39.7)	136 (25.7)	232 (30.1)
Unknown	107 (44.2)	309 (58.4)	416 (54.0)
HIV disclosure (overall)			
No	22 (9.1)	47 (8.9)	69 (8.9)
Yes	220 (90.9)	482 (91.1)	702 (91.1)
HIV disclosure to spouse/partner			
No	141 (58.3)	285 (53.9)	426 (55.3)
Yes	79 (32.6)	197 (37.2)	276 (35.8)
Unknown	22 (9.1)	47 (8.9)	69 (8.9)
ART adherence			
Moderate to very poor	8 (3.3)	38 (7.2)	46 (6.0)
Good to very good	229 (94.6)	482 (91.1)	711 (92.2)
Unknown	5 (2.1)	9 (1.7)	14 (1.8)

**Fig. 1:**
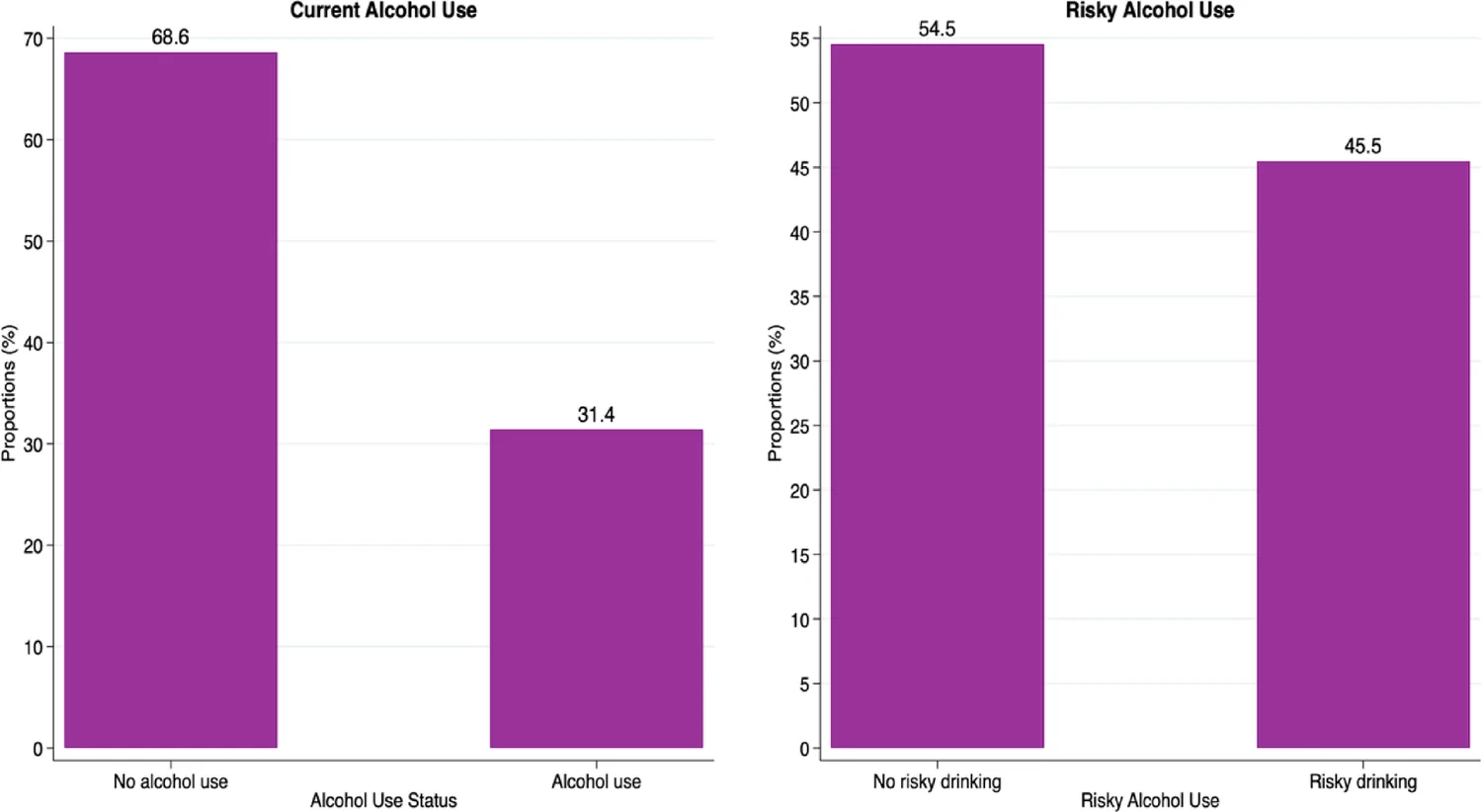
Prevalence of alcohol use and risky alcohol use in the past 12 months. *Risky alcohol use was computed among participants who reported alcohol use in the past 12 months

**Fig. 2:**
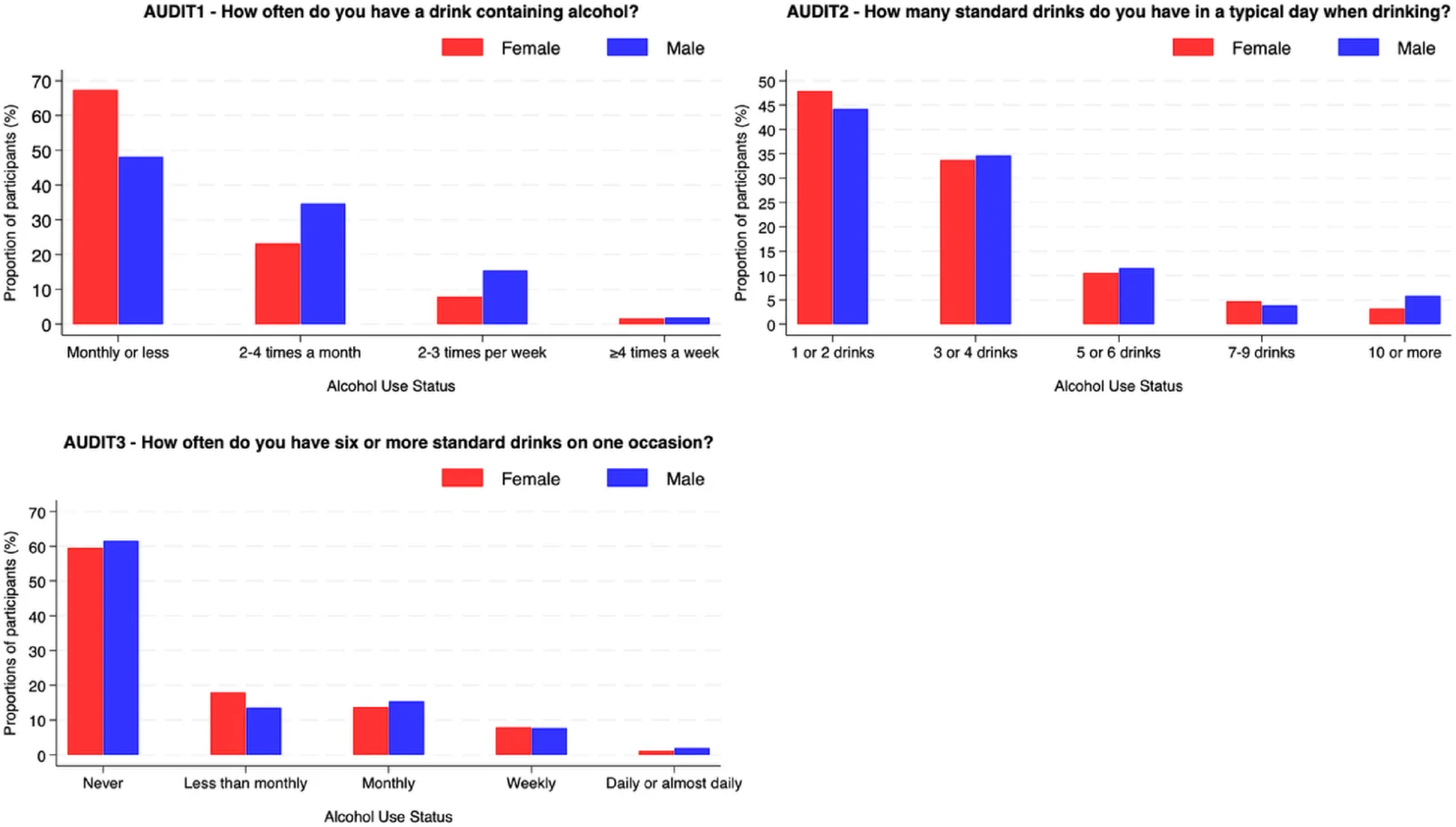
Proportion of participants reporting different alcohol use pattern by for the three AUDIT-C questions. *Proportions are row percentages

About one-third of PLHIV reported current alcohol use (31.4%, *n* = 242). Of those who reported current alcohol use, 45.5% (110/242) were engaging in risky alcohol use. The proportion of risky alcohol use was 14.3% among all participants (110/771) (Fig. 1). One-fifth of participants reported taking alcohol monthly or less (19.8%, *n* = 153). The majority of participants reported taking one to two standard drinks on a typical day when drinking (47.1%, *n* = 114), and 60% had not consumed six or more drinks on one occasion. However, about a quarter (23.2%, *n* = 56) were classified as engaging in HED in the last 12 months; the proportion of participants with HED was slightly higher for males than females (25.0% vs. 22.6%). Generally, males tend to consume alcohol more frequently; however, the pattern of consumption in a typical day is somewhat similar between males and females. (Table 2; Fig. 2).

**Table 2:** Descriptive results of the responses to AUDIT-C questions

AUDIT-C question	*n* (%)
How often do you have a drink containing alcohol?	
Does not take drinks containing alcohol	529 (68.6)
Monthly or less	153 (19.8)
2–4 times a month	62 (8.1)
2–3 times per week	23 (3.0)
≥4 times a week	4 (0.5)
How many standard drinks do you have in a typical day when drinking	
1–2 drinks	114 (47.1)
3 or 4 drinks	82 (33.9)
5 or 6 drinks	26 (10.7)
7–9 drinks	11 (4.6)
10 or more	9 (3.7)
How often do you have six or more standard drinks on one occasion	
Never	145 (59.9)
Less than monthly	41 (16.9)
Monthly*	34 (14.1)
Weekly*	19 (7.9)
Daily or almost daily*	3 (1.2)

*Classified as heavy episodic drinkers (HED) in the last 12 months

### Factors Associated with Risky Alcohol Use

We then examined predictors of risky alcohol use among PLHIV who reported alcohol use in the last 12 months. In univariate analyses, age was the only factor associated with risky alcohol use. The risk of engaging in risky alcohol use decreased with age; participants aged 49 years and above had a lower risk of risky alcohol use compared to participants in the 18–24 age band. In the multivariable model, older age was associated with lower risk of risky alcohol use (RR = 0.27; 95% CI: 0.13–0.54) (Table 3). The risk of risky alcohol use was borderline lower among male (RR = 0.68; 95% CI: 0.46–1.01) compared to female participants. Participants who disclosed their status to their partners were more likely to engage in risky alcohol use compared to those who did not disclose (RR = 1.33; 95% CI: 1.00–1.78).

**Table 3:** Factors Associated with Risky Alcohol Use among adults living with HIV reporting current alcohol use (*N* = 242)

Variables	Univariate RR (95% CI)	*p*-value	Multivariable RR (95% CI)	*p*-value
Sex				
Female	Ref		Ref	
Male	0.76 (0.52–1.12)	0.171	0.68 (0.46–1.01)	0.056
Age groups (years)				
18–24	Ref		Ref	
25–49	0.61 (0.40–0.93)	0.023	0.39 (0.26–0.59)	< 0.001
>49	0.44 (0.22–0.89)	0.022	0.27 (0.13–0.54)	< 0.001
Education level				
No formal education	Ref			
Preschool to college	0.91 (0.51–1.63)	0.758		
Marital status				
Single/never married	Ref			
Currently married/living with partner	1.24 (0.88–1.74)	0.212		
Previously married/lived with partner	1.06 (0.71–1.59)	0.784		
Employment status				
Currently working	Ref			
Not currently working	0.94 (0.56–1.57)	0.807		
Duration since HIV diagnosis				
< 2 years	Ref		Ref	
2–5 years	0.65 (0.39–1.10)	0.109	0.74 (0.42–1.28)	0.283
> 5 years	0.97 (0.60–1.56)	0.899	1.01 (0.61–1.68)	0.961
Duration on ART				
< 6 months	Ref			
6–12 months	0.68 (0.32–1.44)	0.309		
> 12 months	0.77 (0.49–1.21)	0.254		
CD4 counts (cells/µL)				
< 200	Ref			
200-<500	1.30 (0.47–3.57)	0.611		
>=500	1.34 (0.52–3.48)	0.542		
HIV disclosure (overall)				
No	Ref			
Yes	1.47 (0.79–2.76)	0.228		
HIV disclosure to spouse				
No	Ref		Ref	
Yes	1.13 (0.85–1.51)	0.390	1.33 (1.0–1.78)	0.053
Self-reported ART adherence				
Good to very good	Ref		Ref	
Poor to moderate	1.09 (0.54–2.21)	0.810	1.28 (0.67–2.43)	0.455

Somewhat similar results were observed in the second model assessing risky alcohol use among all adults living with HIV (*N* = 771). In this model, the risk of risky alcohol use was higher among the 25–49 (RR: 2.42; 95% CI: 0.90–6.52) and > 49 (RR: 1.72; 95% CI: 0.86–3.47) age bands; however, the 95% CI were wide and crossed the null. Of note, employment status remained significantly associated with risky alcohol use in this model. Participants with no employment were less likely to engage in risky alcohol use (RR: 0.50; 95% CI: 0.26–0.97) compared to those with employment. (Supplementary Table 2).

## Discussion

In our study population of adults living with HIV in urban Tanzania, about one-third of participants reported current alcohol use, and among them, 45.5% were engaged in risky alcohol use. Generally, males consumed alcohol more frequently, but the pattern of consumption in a typical day was somewhat similar between males and females. The majority of participants who reported alcohol drinking in the last 12 months also reported consuming one to two standard drinks on a typical drinking day, with approximately one out of every four participants engaging in HED. Older age was associated with a reduced likelihood of risky alcohol use. Male participants demonstrated a borderline lower risk of engaging in risky drinking compared to females. Additionally, individuals who disclosed their HIV status to their partners were more likely to report risky alcohol use than those who had not disclosed it.

Our findings reflect a higher proportion of current alcohol use for adults living with HIV compared to the urban Tanzanian population. Current alcohol use in urban Dar es Salaam, Tanzania, among the general population was reported to be 17.8% in 2009 [29]. The prevalence of current alcohol reported in our study is also similar to that previously reported among PLHIV in northwestern regions of the country (29.3%) [26]. Compared to our findings, studies in similar SSA settings reported diverse findings on the burden of current alcohol use among PLHIV, ranging between 1% in Nigeria and 66% in Cameroon [36]. Of note, our findings reported a relatively higher burden of risky alcohol use among participants who reported current alcohol use compared to findings from rural regions [26]. The observed difference contrasts with previous evidence that argues for higher alcohol use and alcohol-related harms in rural as compared to urban settings [37, 38], which warrants further investigation to determine drivers of rural-urban disparities in reporting risky alcohol use in Tanzania.

Our estimates are higher than the meta-analytic global prevalence of AUD reported at 29.6% in 2019, with a prevalence of 24.5% in developing countries [3]. Studies in African countries reported a prevalence of 22.0%, with the highest burden being in South Africa (28.8%) [17]. Our higher estimates could also be explained by the increasing prevalence of risky alcohol use over time, as reported elsewhere, which increases the risk of developing AUD [17]. The high burden of risky alcohol use also supports the finding that using alcohol among PLHIV is associated with a greater risk of progressing to harmful drinking or HED than the general population, as reported in several other SSA settings [6]. The pattern of HED reported in our study corroborates these findings. Several studies in similar settings reported a significantly higher proportion of HED among PLHIV, with Zambia having the highest proportion at 82% [6]. Moreover, most of these proportions were significantly higher when compared to the local data on the HED among specific countries’ general population.

In our study, younger adults living with HIV were more likely to engage in risky alcohol use compared to their older counterparts. Similar findings have been reported both among PLHIV and the general population. These differences are often driven by peer influence, lower perceived risk of adverse outcomes, and social norms [39, 40]. Compared to older individuals, young adults are more likely to perceive and experience stigma, especially during transition to adult HIV care, further increasing their risk of maladaptive coping strategies [41].

In this study, disclosure of HIV status to a sexual partner was associated with a higher likelihood of risky alcohol use, with those who disclosed exhibiting a 33% increased risk compared to those who did not. Although HIV status disclosure is generally viewed as beneficial for social support and engagement in care, this finding underscores the complex psychosocial consequences that may follow disclosure. Previous studies have shown that disclosure can be accompanied by heightened stress, fear of negative partner reactions, relationship conflict, or experienced stigma, all of which may increase vulnerability to maladaptive coping strategies such as risky alcohol use [42, 43].

Our study has several limitations. First, we had a relatively small sample in estimating associations with risky alcohol use. AUDIT-C questions for assessing risky alcohol use were administered to only 42% of the trial participants, hence only this sample could be analysed. Since the majority of the study participants were females, males were somewhat under-represented, and hence our findings should be interpreted in the light of this. There is also potential underreporting among participants who responded to alcohol screening questions due to social desirability. This calls for the use of objective measures such as alcohol biomarkers in clinical settings. Due to participant linking difficulties across several trial databases, there is considerable missingness in some of the variables, and only a subset of the trial participants could be analysed in this paper [27]. Furthermore, our study was conducted in urban Tanzania, and therefore, the results may not be generalizable to other settings in SSA.

## Conclusion

Overall, our study reveals a significant burden of current and risky alcohol use, and HED among PLHIV in the largest urban region in Tanzania, with half of the current users presenting with risky drinking. Higher trends of risky alcohol use were also observed among young adults compared to older age groups. Despite any alcohol use being higher in men, females were at a higher risk of engaging in risky alcohol use. Development and evaluation of interventions and strategies targeting alcohol use and risky alcohol drinking among PLHIV in primary care settings are of importance in Tanzania and similar SSA settings. Tailored interventions should aim to address the specific psychosocial and biological risks that may render women and young adults more vulnerable to risky alcohol drinking.

## Supplementary Information

Below is the link to the electronic supplementary material.

Supplementary Material 1

## Data Availability

The datasets used and/or analyzed during the current study are publicly available through the following link. https://dataverse.harvard.edu/dataset.xhtml?%20persistentId=doi:10.7910/DVN/SUVTU8>.
