# Supplementary material for: Alcohol Use, Risky Alcohol Use, and Associated Factors Among Adults Living with HIV in Urban Dar es Salaam, Tanzania
Source: AIDS Behav. 2026 Mar 10;30(10):3008–17. doi: 10.1007/s10461-026-05094-6 (PMC13549399; doi:10.1007/s10461-026-05094-6)
Supplement: Supplementary file 1 [file 10461_2026_5094_MOESM1_ESM.docx]

**Supplementary Table 1: Comparison of participants with outcome data and those missing outcome data**

| **Variable** | **Has outcome data (N=771)** | **Missing outcome data (N=1,068)** | **Total**  **N=1,839** | **Pearson chi-square** | **p-value** |
| --- | --- | --- | --- | --- | --- |
| **Sex** |  |  |  | 1.1406 | 0.286 |
| Female | 636 (82.5%) | 860 (80.5%) | 1,496 (81.3%) |  |  |
| Male | 135 (17.5%) | 208 (19.5%) | 343 (18.7%) |  |  |
| **Age groups** |  |  |  | 2.850 | 0.240 |
| 18-24 | 31 (4.0%) | 28 (2.6%) | 59 (3.2%) |  |  |
| 25-49 | 641 (83.1%) | 898 (84.1%) | 1,539 (83.7%) |  |  |
| >49 | 99 (12.8%) | 142 (13.3%) | 241 (13.1%) |  |  |
| **Education level** |  |  |  | 2.540 | 0.281 |
| None | 33 (4.3%) | 33 (3.1%) | 66 (3.6%) |  |  |
| Preschool to Secondary | 720 (94.1%) | 1,015 (95.8%) | 1,735 (95.1%) |  |  |
| Post Secondary/College | 12 (1.6%) | 12 (1.1%) | 24 (1.3%) |  |  |
| **Marital status** |  |  |  | 35.368 | <0.001 |
| Single/Never Married | 218 (28.6%) | 192 (18.2%) | 410 (22.5%) |  |  |
| Currently Married/Living with Partner | 371 (48.6%) | 523 (49.5%) | 894 (49.1%) |  |  |
| Previous Married/Lived with Partner | 174 (22.8%) | 341 (32.3%) | 515 (28.3%) |  |  |
| **Employment status** |  |  |  | 2.362 | 0.307 |
| Yes | 658 (85.3%) | 901 (84.4%) | 1,559 (84.8%) |  |  |
| No | 113 (14.7%) | 164 (15.4%) | 277 (15.1%) |  |  |
| 99 | 0 (0.0%) | 3 (0.3%) | 3 (0.2%) |  |  |
| **Duration since HIV diagnosis** |  |  |  | 0.905 | 0.636 |
| <5 years | 245 (67.5%) | 324 (66.5%) | 569 (66.9%) |  |  |
| 5-10 years | 78 (21.5%) | 116 (23.8%) | 194 (22.8%) |  |  |
| >10 years | 40 (11.0%) | 47 (9.7%) | 87 (10.2%) |  |  |
| **Duration on ART** |  |  |  | 4.543 | 0.103 |
| <6 months | 66 (14.0%) | 67 (10.2%) | 133 (11.8%) |  |  |
| 6-12 months | 39 (8.3%) | 67 (10.2%) | 106 (9.4%) |  |  |
| >12 months | 366 (77.7%) | 521 (79.5%) | 887 (78.8%) |  |  |
| **CD4 counts (cells/µL)** |  |  |  | 0.384 | 0.825 |
| <200 | 32 (9.0%) | 83 (8.7%) | 115 (8.8%) |  |  |
| 200-<500 | 91 (25.6%) | 261 (27.3%) | 352 (26.9%) |  |  |
| >=500 | 232 (65.4%) | 611 (64.0%) | 843 (64.4%) |  |  |
| **HIV disclosure (Overall)** |  |  |  | 0.002 | 0.968 |
| No | 69 (8.9%) | 95 (8.9%) | 164 (8.9%) |  |  |
| Yes | 702 (91.1%) | 973 (91.1%) | 1,675 (91.1%) |  |  |
| **HIV disclosure to spouse** |  |  |  | 2.108 | 0.147 |
| No | 426 (60.7%) | 556 (57.1%) | 982 (58.6%) |  |  |
| Yes | 276 (39.3%) | 417 (42.9%) | 693 (41.4%) |  |  |
| **ART adherence** |  |  |  | 2.130 | 0.144 |
| Poor to Very Poor | 46 (6.1%) | 48 (4.5%) | 94 (5.2%) |  |  |
| Good to Very Good | 711 (93.9%) | 1,010 (95.5%) | 1,721 (94.8%) |  |  |

*The comparison in this table excludes missing values in the individual covariates

**Supplementary Table 2: Factors Associated with Risky Alcohol Use among all adults living with HIV (N=771)**

| **Variables** | **Univariate RR (95% CI)** | **p-value** | **Multivariable RR (95% CI)** | **p-value** |
| --- | --- | --- | --- | --- |
| **Sex** |  |  |  |  |
| Female | Ref |  | Ref |  |
| Male | 0.98 (0.62 - 1.56) | 0.944 | 0.99 (0.61 - 1.56) | 0.950 |
| **Age groups (years)** |  |  |  |  |
| 18-24 | Ref |  | Ref |  |
| 25-49 | 0.77 (0.37 - 1.62) | 0.498 | 2.42 (0.90 - 6.52) | 0.081 |
| >49 | 0.42 (0.16 - 1.11) | 0.080 | 1.72 (0.86 - 3.47) | 0.126 |
| **Education Level** |  |  |  |  |
| No formal education | Ref |  |  |  |
| Preschool to College | 0.78 (0.37 - 1.65) | 0.517 |  |  |
| **Marital Status** |  |  |  |  |
| Single/Never Married | Ref |  |  |  |
| Currently Married/Living with Partner | 1.07 (0.71 - 1.64) | 0.740 |  |  |
| Previously married/lived with partner | 1.12 (0.69 - 1.83) | 0.642 |  |  |
| **Employment status** |  |  |  |  |
| Currently working | Ref |  | Ref |  |
| Not currently working | 0.52 (0.27 - 1.00) | 0.049 | **0.50 (0.26 - 0.97)** | **0.040** |
| **Duration since HIV diagnosis** |  |  |  |  |
| <2 years | Ref |  | Ref |  |
| 2-5 years | 0.72 (0.38 - 1.35) | 0.309 | 0.69 (0.37 - 1.31) | 0.259 |
| >5 years | 0.83 (0.45 - 1.54) | 0.565 | 0.80 (0.43 - 1.50) | 0.492 |
| **Duration on ART** |  |  |  |  |
| <6 months | Ref |  |  |  |
| 6-12 months | 0.85 (0.35 - 2.07) | 0.715 |  |  |
| >12 months | 0.71 (0.40 - 1.26) | 0.238 |  |  |
| **CD4 T-cell (counts/ml)** |  |  |  |  |
| <200 | Ref |  |  |  |
| 200-<500 | 1.52 (0.46 - 5.00) | 0.487 |  |  |
| >=500 | 1.98 (0.65 - 6.00) | 0.229 |  |  |
| **HIV Disclosure (overall)** |  |  |  |  |
| No | Ref |  |  |  |
| Yes | 1.45 (0.70 - 2.98) | 0.318 |  |  |
| **HIV Disclosure to spouse** |  |  |  |  |
| No | Ref |  | Ref |  |
| Yes | 0.99 (0.68 - 1.41) | 0.914 | 0.98 (0.68 - 1.43) | 0.933 |
| **Self-reported ART adherence** |  |  |  |  |
| Good to Very Good | Ref |  | Ref |  |
| Poor to Moderate | 0.59 (0.23 - 1.53) | 0.276 | 0.57 (0.22 - 1.48) | 0.250 |
